# Integrated mental health care in a multidisciplinary maternal and child health service in the community: the findings from the Suzaka trial

**DOI:** 10.1186/s12884-019-2179-9

**Published:** 2019-02-06

**Authors:** Yoshiyuki Tachibana, Noriaki Koizumi, Chikako Akanuma, Hiromi Tarui, Eizaburo Ishii, Tomomi Hoshina, Ayuko Suzuki, Akiko Asano, Shiho Sekino, Hiroto Ito

**Affiliations:** 1 Division of Infant and Toddler Mental Health, Department of Psychosocial Medicine, National Center for Child Health and Development, Tokyo, Japan; 2 Nagano Prefectural Public Health Center for Mental Health, Nagano, Japan; 3 Suzaka City Public Health Center, Nagano, Japan; 4 Department of Pediatrics, Nagano Prefectural Suzaka Hospital, Nagano, Japan; 5 Research Center for Overwork-Related Disorders, National Institute of Occupational Safety and Health, Japan Organization of Occupational Health and Safety, Kanagawa, Japan; 6 Department of Pediatric Palliative Care, Shinsei Hospital, Nagano, Japan; 7 Nagano Nursing Association, Nagano, Japan

**Keywords:** Mental health, Postnatal depression, Mother, Pregnant, Continuum supports, Pregnancy periods, Multidisciplinary

## Abstract

**Background:**

Perinatal mental health problems such as mood disorders are common. We propose a new multidisciplinary health service intervention program providing continuous support to women and their children from the start of pregnancy till after childbirth. The aim of this study was to examine the effects of the program with respect to making women’s mental health better in the postpartum period and improving the state of care for women and their children in the perinatal period.

**Methods:**

We performed a controlled study to investigate the effectiveness of the program in Suzaka City, Japan. The women’s mental health status was assessed using the Edinburgh Postnatal Depression Scale (EPDS) 3 months postpartum. Of 349 women, 210 were allocated to the intervention group and 139 to the control group. From April 2014 to March 2015, the number of the pregnant women who were followed-up by the multidisciplinary meeting in the intervention and control groups were 60 and 4, respectively. In the same period, the number of the pregnant women who were identified as requiring intensive care were 21 and 2, respectively.

**Results:**

The total EPDS score, which was the primary outcome of the present study, differed significantly between the intervention and control groups (Mean [SD] = 2.74 (2.89) and 4.58 [2.62], respectively; *p* < 0.001). The number of the women receiving counseling from a public health nurse (5.3% in intervention group, 0.7% in control group, *p* = 0.02), attending maternal seminars (attendant ratio: 46% whereas 16%, *p* = 0.01), and receiving home visits by public health nurses (home visit ratio: 93.8% whereas 82.6%, *p* < 0.001) was significantly higher in the intervention group compared to the control group.

**Conclusions:**

The present study indicates that continuum support provided by integrated mental health care through a multidisciplinary maternal and child health service in the community can make women's mental health better in the postpartum period and help women and their children receive more health services from public health nurses.

**Trial registration:**

Name of registry: Research for the effectiveness of a multi-professional health service intervention program of continuum supports for mother and child which starts for pregnancy periods to enhance maternal mental health.

UMIN Clinical Trials Registry number: UMIN000032424.

Registration date: April 29th, 2018.

Registration timing: retrospective.

**Electronic supplementary material:**

The online version of this article (10.1186/s12884-019-2179-9) contains supplementary material, which is available to authorized users.

## Background

Mental health problems such as mood disorders are common among women in their perinatal period [1], and can negatively affect their daily quality of life, their relationship with the child [2], as well as the child’s behavior [3] and development [3–5].

Many clinical guidelines recommend antenatal mental health screening [5–9], although the evidence on the efficacy of such measures in improving outcomes as well as the perinatal mental health pathways in routine clinical practice is still limited [8, 10]. The World Health Organization suggests that effective screening programs should have an adequate understanding of the condition; a simple, safe, validated screening test; effective treatment for those screened as positive; and adequate resources to ensure that the programs are implemented in an acceptable and expert manner [11].

Various psychosocial interventions targeting women in antenatal periods have been developed [12, 13]. In Japan, public health nurses perform home visits within 4 months postpartum. However, additional support provided continuously by public health nurses and other related professionals from the start of pregnancy to the child-rearing stage may be beneficial. There are currently no evidence-based standard multidisciplinary health service intervention programs providing such support to the mother and child. Therefore, there is a need to assess existing perinatal networks to examine the effectiveness of different health service intervention programs in delivering care as suggested through “Research recommendations” in the National Clinical Guidelines of the National Institute for Health and Care Excellence [7].

The current paper proposes a new integrated multidisciplinary health service intervention program aimed to provide mental health care to the mother and child throughout pregnancy and childbirth. The hypotheses, which was that the program would make women’s mental health better in the postpartum period and improve the state of care for the women and their children, was tested using a controlled study that investigated the effectiveness of a multidisciplinary health service intervention program that provided continuous support to women and their children in Suzaka City, Japan.

## Methods

### Study design

This study was a controlled trial comparing a new multidisciplinary health service intervention program providing continuous support to the mother and child from the start of pregnancy (Suzaka Program: the intervention) to the usual care protocol (used prior to the start of the Suzaka Program: the control).

The effectiveness of the program was assessed by measuring the women’s mental health condition using the Edinburgh Postnatal Depression Scale (EPDS) 3 months postpartum. Both groups received home visits from public health nurses during the postnatal periods, and were asked to continue with their usual daily lives throughout the pregnancy and postnatal periods. Only the women in the intervention group received interviews at the time of submitting their pregnancy notification sheets to the Suzaka City Office. Additionally, they also received follow-up visits from the public health nurses if necessary.

### Subjects

**Fig. 1:**
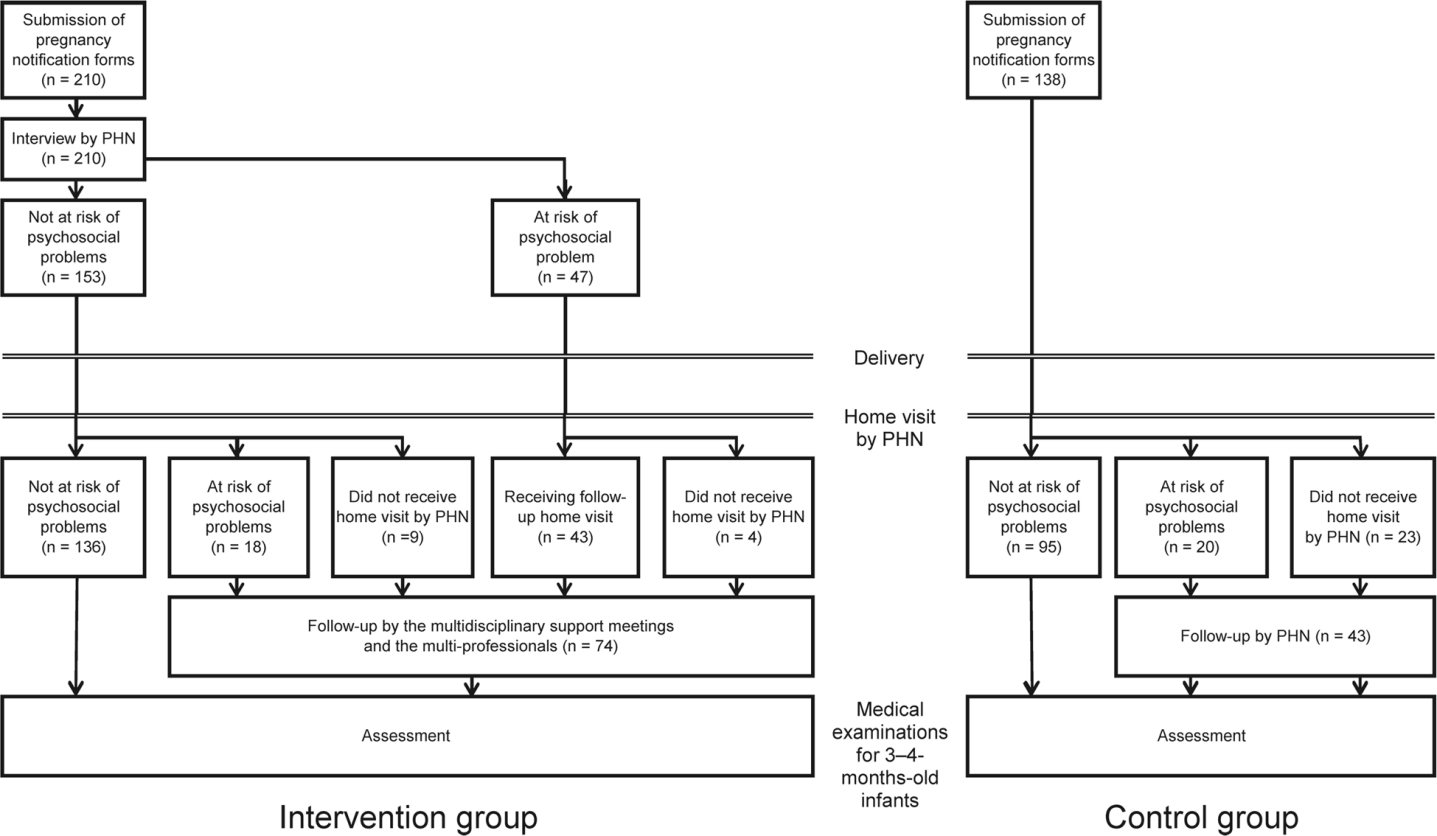
Study flowchart. Foot notes: PHN - public health nurse; PHC - public health center; * indicates 19 mothers thought to be at risk of psychosocial problems at the time of neonatal home visits by public health nurses (9 mothers did not receive neonatal home visits by the public health nurses); ** indicates 23 out of 138 mothers that did not receive neonatal home visits by the public health nurses; *** indicates 20 out of 115 mothers that were thought to be at risk of psychosocial problems and were followed up by the public health nurses

This study included pregnant women from Suzaka City in the Nagano prefecture of Japan, and the inclusion criterion was all women who submitted their pregnancy notification sheets to the Suzaka City Office. There was no exclusion criterion for this study. After obtaining informed consent, a public health nurse (CA) selected specifically for this program interviewed the women using an interview sheet. This data was then entered into the system by a different person. Figure 1 shows a flowchart of the current study. This study included 348 women, of which 138 had submitted their pregnancy notification forms between June 2013 and March 2014 and were allocated to the control group. The remaining 210 women who had submitted their forms between April 2014 and July 2014 were allocated to the intervention group. Those who were identified as being at risk of psychosocial problems were included in multidisciplinary support meetings once a month. In multidisciplinary support meetings, which were held once a month, the care plan for women who were identified as being at risk for developing psychosocial problems was discussed. The multidisciplinary team meeting included public health nurses, obstetricians, midwives, nurses, medical social workers, and psychiatrists. The total number of professionals attending these meetings ranged from 15 to 25.

### The intervention program

**Fig. 2:**
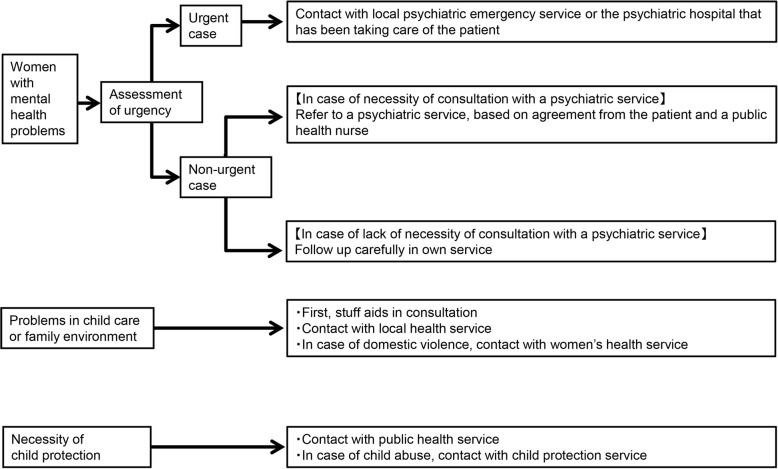
Referral and management protocols for services for women and children with psychosocial problems. Foot notes: In case of women with a past history or familial history of severe psychiatric disorders such as major depressive disorder and bipolar disorder, the occurrence of psychiatric symptoms was assessed and the women were referred to psychiatric services if necessary. “Urgent case” indicates i) strong suicidal ideas or intention of self-harm that cannot be self-managed; ii) sudden emergence of psychotic symptoms; iii) risk of self-harm or causing harm to others

**Fig. 3:**
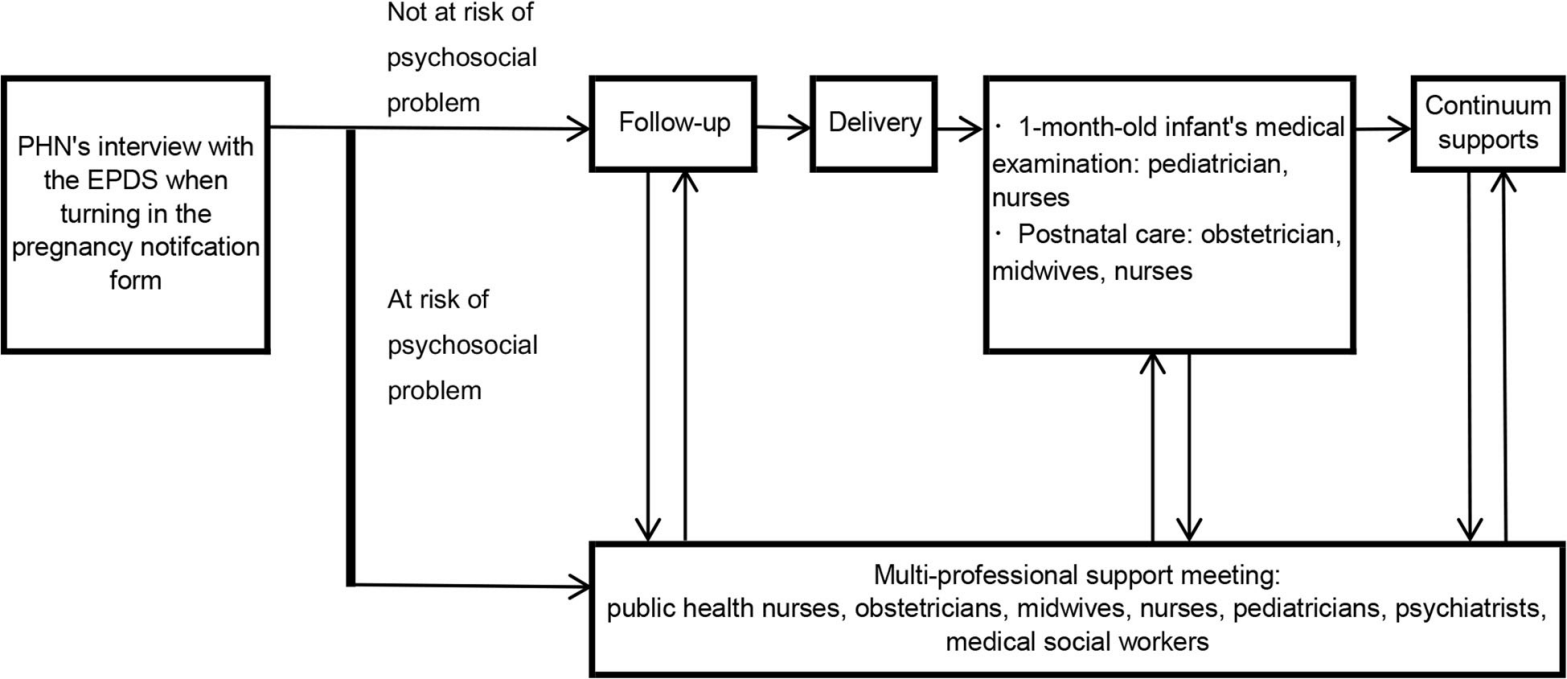
Shema of Suzaka Program’s continuum and multidisciplinary maternal and child health service. Footnote: PHN - public health nurse; EPDS - Edinburgh Postnatal Depression Scale

This program commenced with support from a public health nurse when the women submitted their pregnancy notification forms to the local government office, and aimed to include as many women as possible. Upon submission of the pregnancy notification form, a public health nurse specifically chosen for this program carried out routine interviews with the women. They were informed that the data from these interviews would be shared with related professionals involved in maternal and child health care to aid in the development of customized care plans, and written consent was collected from all participants. Thereafter, the women answered the psychosocial screening questionnaire. The public health nurses interviewed the women on whether they had any other psychosocial problems using the psychosocial screening questionnaire, with the aim of developing a relationship between the Suzaka City public health service and the pregnant women and making it easier for them to utilize the public health services if they had any concerns about the pregnancy and child care. The psychosocial assessment sheet included the Japanese version of the Edinburgh Postnatal Depression Scale [14, 15], as well as the risk factors of postnatal depression identified by a Japanese epidemiological study [16]. The public health nurses carefully followed up the women based on the results of the interviews. The intervention program, developed by two psychiatrists (YT and NK) and two public health nurses (CA and HT) from Suzaka City [16], program aimed to provide continuous support to the mother and child from the start of pregnancy and after childbirth. In keeping with the NICE guidelines [17], it consisted of a multidisciplinary clinical network which had the following four features: a) it provided multidisciplinary perinatal services, including consultation and advice from maternity, mental health and community services; b) pregnant women and breast-feeding women could access advice on the risks and benefits of consuming psychotropic medication during the perinatal periods from psychiatrists and obstetricians; c) it had clear referral and management protocols for services across all levels of the existing stepped-care framework for women and children with psychosocial problems [18, 19] [attention was given to assessment of urgency, family environment issues, and the necessity for child protection and, if deemed necessary, the intervention team contacted other relevant professionals and provided multidisciplinary support (Fig. 2)]; and d) the Suzaka program provided care for service users by defining roles and competencies for all professional groups involved, thus enabling provision of continuous care for the mother and child (Fig. 3).

The multidisciplinary support meetings, which were held for the women of the intervention group, were also an important characteristic of the Suzaka program. These meetings, which were held once a month in the Nagano Prefectural Suzaka Hospital, were attended by the Suzaka City public health nurses, obstetricians, midwives, nurses, medical social workers, pediatricians, and psychiatrists. The public health nurses identified the women and children with psychosocial problems requiring follow-up, and discussed their management plan at the multidisciplinary support meetings. The team members discussed how to support the women and their families and developed individual care plans customized to the women and their families’ needs. The multidisciplinary meetings were developed based on a hierarchical model [20], which has been suggested to be most appropriate for a perinatal mental health network [17] (see Additional file 1). A public health nurse from the Suzaka City Office selected specifically for this program acted as the manager and clearly specified and delegated responsibilities. In cases requiring child protection or intensive child support, they collaborated with the local child protection center. Tailor-made care plans were developed for the women and their families. The care plans were also updated in the public health center meetings through follow-ups by the public health nurses; further, these plans were repeatedly discussed in the multidisciplinary support meetings (See Additional file 2 for a case management example of the multidisciplinary continuum support network).

### Assessment tests

#### The intervention group

The women in the intervention group were asked to complete a psychosocial questionnaire (see Additional file 3) at the time of submitting their pregnancy notification form to the local government office. The questionnaire was developed based on known predictors of postnatal depression and the standard obstetrical interview sheets in Japan. Additionally, the risk factors identified by Robertson [10], O’Hara [1], and our previous epidemiological study conducted in the Setagaya Ward in Japan [21] were also included. The women were classified based on their risk of PND (high-risk or not) using the cut-off scores of the Japanese version of the EPDS (at 8/9) [15].

We assessed mother’s mental health when the infant was 3–4-months-old through medical examinations at public health centers (T2) using the EPDS. The questionnaire consisted of psychosocial questions as well as the Japanese version [17] of the EPDS [18]. The women were also asked what services they and their children had received from the start of their pregnancy up to that point in time (See Additional file 4).

#### The control group

We assessed mother’s mental health when the infant was 3–4-months-old through medical examinations at public health centers (T2) using the EPDS with the women of the control group. Information on the persons they lived with, history of smoking before and after pregnancy, history of drinking before and after pregnancy, medications taken during pregnancy, and history of psychiatric and physical illness treatment were also collected.

### Outcomes

The primary outcome of this study was the EPDS total score during the 3–4 months postpartum period. The secondary outcomes were the number of women who were followed up by the multidisciplinary meetings, the woman who required intensive care, counseling by the public health nurses, attendance at a maternal seminar, postnatal care usage, home visits, telephone counseling for child care, counseling for child care at a public health center, and family support usage.

### Main analysis

Two sample t-tests were used to examine differences in the women’s ages between the intervention and control groups, while the effectiveness of the program was examined by comparing the outcomes of the two groups using the Student’s t-tests.

### Sub-analysis

We performed two stratified analyses in which the participants were classified into two groups: “primipara and multipara” and “participants with a history psychiatric treatment and participants without a history of psychiatric treatment”. Hence, primipara means those who delivered the first offspring while participating in this study; multipara means those who had delivered once or more before participating in this study. The variables that were analyzed included risk factors for antenatal depression that were reported in our previous study [16].

Data were reported as “the mean (standard deviation)”. Statistical significance was set at *p* < 0.05, and all data analyses were performed using SPSS version 22.0 J for Windows (SPSS Inc., Tokyo, Japan).

## Results

The mean ages of the women in the control and intervention groups were 30.86 (0.28) and 30.50 (0.26) years, respectively; this difference was not statistically significant (*p* = 0.86). Regarding the pregnancy stage, women in the intervention and control groups were enrolled at a mean of 10.80 (4.27) and 10.50 (4.41) weeks, respectively. The patient demographics by group have been shown in Table 1. The results of the psychosocial questionnaire of the intervention group’s participants have been shown in Additional file 5.

**Table 1:** Basic characteristics of the participants of the intervention group

	Missing	Intervention group (*n* = 210)	Missing	Control group (*n* = 138)
Persons who live with				
partner	1	205	1	137
child				
0		91		55
1		84		58
2		25		17
3 or more		10		8
partner’s father		26		19
partner’s mother		33		23
mother		25		18
father	1	19		14
siblings				
0		196		128
1		10		7
2 or more		4		3
others		15		9
Smoking				
Non-smoker also before pregnancy		169		109
Stopped after pregnancy		37		26
Yes		4		3
Drinking	1		1	
Non-drinker also before pregnancy		104		65
Stopped after pregnancy		105		62
Medication	4		4	
Yes		24		18
No		182		116
History of psychiatric treatment before pregnancy	37		26	
Yes		13		9
No		160		103
History of physical illness treatment before pregnancy	37		26	
Yes		17		12
No		156		100

**Fig. 4:**
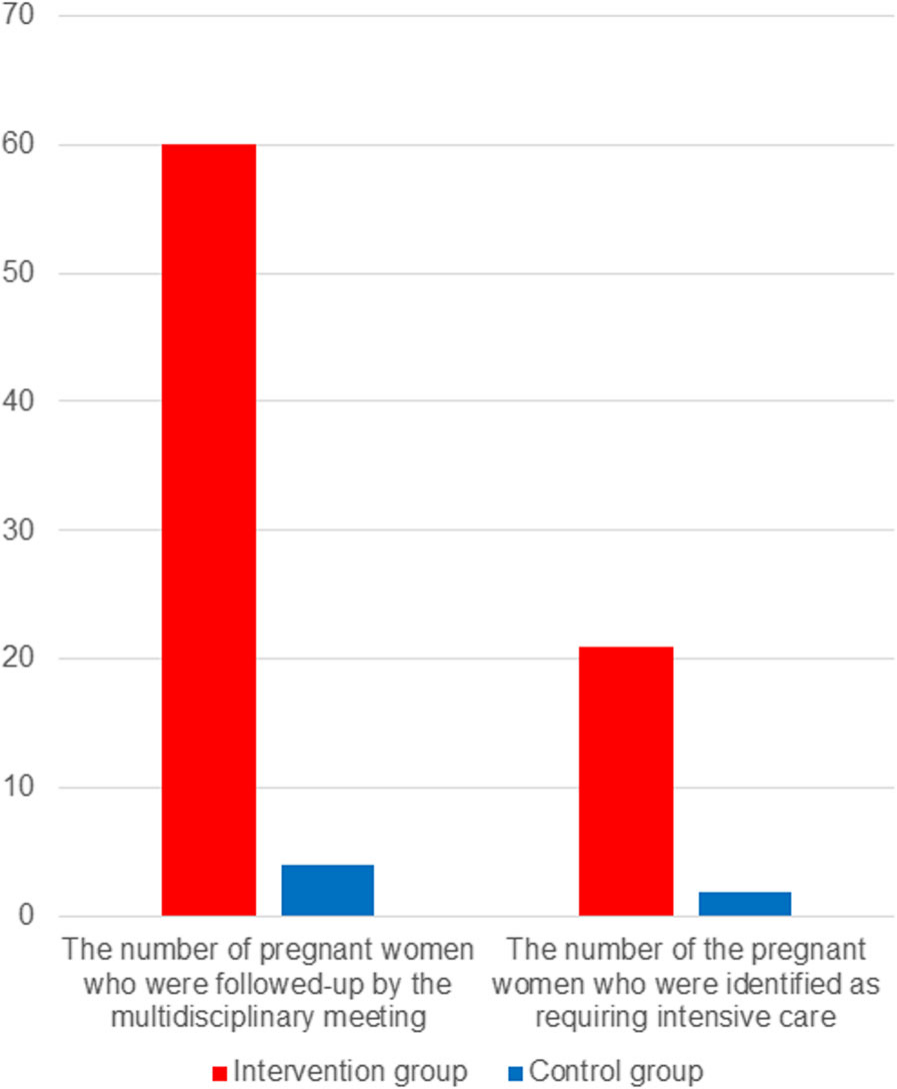
The number of pregnant women followed up by the multidisciplinary meetings and were identified as requiring intensive cares. Footnotes: Year 1 indicates the Japanese fiscal year from April 2014 to March 2015; Year 2 indicates the Japanese fiscal year from April 2013 to March 2014

The primary and secondary outcomes have been shown in Table 2. Between April 2014 and March 2015, the number of pregnant women who were followed-up by the multidisciplinary meeting in the intervention and control groups were 60 and 4, respectively. Over the same period, the number of pregnant women who were identified as requiring intensive care were 21 and 2, respectively (see Fig. 4). Among the secondary outcomes, the ratio of counseling by public health nurses, participation at the maternal seminars, and home visits by the public health nurses increased significantly in the intervention group compared with the control group (*p* = 0.02, 0.01, and < 0.001, respectively). The ratio of postnatal care usage, telephone counseling for child care, counseling for child care at public health center, and family support usage between the two groups did not differ significantly (see Table 2). The total EPDS score, which was the primary outcome of the present study, differed significantly between the intervention [2.74 (2.89)] and control [4.58 (2.62)] groups (*p* < 0.001). The mean EPDS scores of the intervention group, which were obtained at the initial interview by the public health nurses when the women submitted their pregnancy notification forms) and those that were obtained at the neonatal home visits were 3.59 (1.48) and 3.16 (3.32), respectively.

**Table 2:** The comparisons of the service usage between the control and the intervention group

		Intervention group			Control group	
		Mean or Number	SD or %	*p* value	Mean or Number	SD or %
Primary outcome						
Total score of the EPDS		2.74	2.89	< 0.001**	4.58	2.62
Secondary outcomes						
PHN’s interview				< 0.001**		
	Yes	201	96.2		0	0.0
	No	9	4.3		138	100.0
PHN’s counselling				0.02*		
	Yes	11	5.3		1	0.7
	No	199	95.2		137	99.3
maternal seminar				0.01*		
	Yes	46	22.0		16	13.1
	No	164	78.5		122	16.9
postnatal care usage				0.42		
	Yes	11	5.3		4	3.0
	No	199	95.2		134	97.0
home visit				< 0.001**		
	Yes	197	94.3		114	82.6
	No	13	6.2		23	16.7
telephone counselling for child care				0.34		
	Yes	8	3.8		3	2.2
	No	202	96.7		135	97.8
Counselling for child care at PHC				0.22		
	Yes	41	19.6		20	16.9
	No	169	80.9		118	83.1
Family support usage				0.34		
	Yes	1	0.5		2	1.5
	No	209	100.0		136	98.5

SD: standard deviation. EPDS indicates Edinburgh Postnatal Depression Scale, PHN: public health nurse. PHC indicates public health center, p value: the value of the Chi-square test for each variable between the two groups, *: statistically significant (p < 0.05) in the analyses, **: statistically significant (p < 0.001) in the analyses

The results of the sub-analysis are shown in Table 3. This intervention program had significant effects on the EPDS total score at 3–4 months postpartum in both primiparas and multiparas. The results also showed that the intervention program had significant effects on women who did not have a history of psychiatric treatment, but not on women who had a history of psychiatric treatment.

**Table 3:** Comparison of the outcomes in the intervention and control group according to parity and history of psychiatric treatment

		Intervention group		Control group		*P* value
		Number	Mean (SD)	Number	Mean (SD)	
Parity	Primipara	91	2.58 (2.83)	55	4.51 (2.33)	< 0.001**
	Multipara	119	2.94 (2.87)	83	4.63 (2.80)	< 0.001**
History of psychiatric treatment	(−)	192	2.65 (2.57)	129	4.31 (1.81)	< 0.001**
	(+)	18	3.78 (5.20)	9	8.44 (6.84)	0.60

*P* value: The *p* value of the *t*-tests between the intervention and the control group SD: standard deviation

(−): Participants who did not have a history of psychiatric treatment

(+): Participants who had a history of psychiatric treatment

## Discussion

The study demonstrated the effectiveness of a multidisciplinary health service intervention program, which aimed to provide continuous support to the women and their children from the start of pregnancy to childbirth, in making women’s mental health better in the 3–4 months postpartum period, which was measured by the EPDS. It also showed an increase in the number of women and children who were identified as being at risk of psychosocial problems, and provided them with support, counseling from public health nurses, maternal seminars, and home visits.

The significant differences in the EPDS scores of the 3–4-month-old infants’ medical examinations between the intervention and control group suggests that the Suzaka Program could make women’s mental health better in the postnatal period. There are various types of interventions in perinatal periods: health visitors’ psychological intervention training (e.g. [22]; home- based intervention (e.g. [23–25]); interpersonal psychotherapy [26] and applied interventions of its methods [27–29]; educational intervention [30–32]; social supports [33–35]; midwifes’ interventions [36–41]. These intervention program were performed mainly by one profession. On the other hand, the present program has characteristics case-management by multi-professionals. The efficacy of case-management has been demonstrated in the area of suicide prevention [42–44]. In our program, a public health nurse played a role of case manager. She made initial interviews and initial assessments. The multi-professional meetings discussed care plans. In case of necessary, referrals were considered by the case manager and multi-professional meetings. Then, the public health nurses and other professionals gave information for the problem which the women had and offered helpful resources such as baby sitters, home workers if the women needed. The women were followed up by multi-professionals. These processes can lead to continuum care for the women, their children from pregnancy periods [45].

In this program, the public health nurses interviewed and performed mental health screenings in all pregnant women who had submitted their pregnancy notification forms. This may be considered as a mix of a population and high-risk approach toward providing psychosocial support to the women. In addition to acting as a form of mental health screening, we believe that the EPDS interviews conducted by the public health nurses at T1 also played the following roles: 1) opening up the conversation about psychosocial issues; 2) raising awareness and educating pregnant women about the various psychiatric and psychosocial conditions [8]; and 3) developing relationships with the women by inquiring about their psychosocial problems. We think these processes of our program can build up a good relationship between care provider (public health nurses) and women. Such good relationship allows the establishment of trust, open and frank information receiving, in turn, to a better understanding of the needs of the expectant women [22, 46, 47]. This relationship between the women and the public health nurses could lead to an increase in the usage of counseling services provided by the public health nurses as well as maternal seminars. Additionally, it could also have improved the ratio of neonatal home visits between the intervention and control groups, resulting in increased care for mother and child, thereby strengthening intervention effects. These improvements in the secondary outcomes of this study were thought to have contributed to the improvements in the primary outcome. Public health visitors have a pivotal part to play in prevention, early identification, prompt treatment and improved outcomes for mothers, their partners and their babies; i.e. roles in psychosocial assessment, assessing and managing physical and mental health, in assessing the level of contact and support needed, in facilitating access to integrated and collaborative care [17, 48]. In this program, the public health nurses played part as such. We think the present program can show one of the model of the care model which public health nurses played a central role in the multidisciplinary support network.

The multidisciplinary support network played a key role in the improvements observed. Although the obstetric departments in hospitals typically have lots of information about pregnant women, the public health nurses in Japan only receive this information on the women and their children following neonatal home visits. Although many professionals are involved in maternal and child health care, they have limited opportunities to coordinate with professionals in other healthcare organizations, resulting in difficulties associated with referrals to related professionals in cases of special care [16, 17]. Previous studies have revealed that 1.7% of women giving birth were referred to specialists for postnatal depression, and approximately 3 to 5% of women exhibited moderate or severe depression [14, 49]. Because the multidisciplinary support meetings involve various professionals related to maternal and child health services, it is easy to obtain support for mother and child health care through their referrals. In case of the women with mental health problems, because the multidisciplinary meeting involves specialists of mental health services, the care plans derived from the multidisciplinary support meeting are made considering the viewpoints of specialists, and thus appropriate referrals can be obtained when needed.

The results of the stratified analyses suggest that this intervention program may be effective for improving the mental health of both primipara women and multipara women in the 3–4 months postpartum period. Hung’s study suggested that tailored nursing interventions based on differences in parity may help to reduce postpartum stress and help to prevent postnatal psychological problems [50, 51]. In her study, the primiparas had higher scores for postpartum stress, concerns about negative body changes, concerns about maternal role attainment, while multiparas had higher scores than primiparas regarding concerns about the lack of social support [50]. Our intervention program supports those problems and concerns. In cases in which a woman has psychological stress, concerns about her body condition, or does not have self-efficacy as a mother, related professionals such as public health nurses and midwives can support her. When a woman has a lack of social support, various forms of support, including home help services and childcare services will be proposed to her when the related professional notices a lack of social support. These characteristics of our program may be effective for both primipara and multipara women.

There was a discrepancy in the stratified analysis between the women with and without a history of psychiatric treatment. On the other hand, the results of the main analysis and the stratified analyses of the patients without a history of psychiatric treatment were consistent. There are two possible explanations for the discrepancy in the results of the women with and without a history of psychiatric treatment and the results of the stratified analysis of the participants with a history of psychiatric treatment did not show statistically significant effects. The first possibility is that the analysis for the participants with a history of psychiatric treatment lacked the statistical power needed to detect a significant difference between the two groups. The second possibility is that some other intervention effects might have obscured the effects of the intervention program. Since the T1 section of the questionnaire only asked whether the respondent had “a history of psychiatric treatment”, the number of participants who were currently receiving psychiatric treatment among the patients with a history of psychiatric treatment was not clear in either group. However, it is thought that many of the patients with a history of psychiatric treatment were currently receiving psychiatric treatment. Such treatment for women with mental health problems might have obscured the effects of the intervention program. A previous study suggested that psychiatric treatments such as psychoeducation, psychotherapy, and medication have positive effects on women’s mental health during the perinatal period [17, 23]. Thus, the intervention program may be expected to have some effects in women with or without a history of psychiatric treatment. Further research should be performed to investigate the effects of the intervention program on women with a history of psychiatric treatment and their association with the effects of other coexisting interventions.

The current study had several limitations. Firstly, mental health outcome was assessed using the EPDS, and no structured clinical interviews were conducted. Thus, the number of women with clinical depression was not clarified in this study. Secondly, the demographic characteristics of the control group were not assessed, and the baseline data of the two groups were not adjusted for in the analyses. However, the study design and the similarities in the area of residence between the two groups suggest that the demographic characteristics would not exhibit any major differences. Thirdly, since this study did not measure the pre-intervention EPDS (when they turned in the pregnancy notification form), we cannot suggest that this program may improve women’s postnatal mental health, we can only suggest that it may make women’s mental health better in the postnatal period. However, the program for the participants of the intervention group was held in the area in which the control group lived, the baseline EPDS data at the time when they turned in their pregnancy notification form can therefore be predicted to be similar. Thus, although we could not compare the two groups with a pre–post design, we thought the results in relation to the primary outcome of the present study would—to some extent—serve as a reference to infer the effects of the intervention program. Fourthly, this study performed a sub-analysis with parity and history of psychiatric treatment, as these were shown to be important antenatal risk factors for postnatal depression in our previous study [50]. However, there may be other confounding factors affecting the intervention program. Further studies should be performed to investigate the effects of the intervention program on women in the perinatal period who have other confounding factors besides parity and a history of psychiatric treatment. Fifthly, the study sample may not be representative of the entire Japanese population as Suzaka is a small rural city and has only one public health center and one hospital. Therefore, development of a multidisciplinary network was easier in Suzaka City than that in a big city with many public health centers and hospitals. Implementation of the present intervention program in a big city or in other country would require modifications in order to fit the local resources available for mother and child health services. However, we believe help from multidisciplinary collaborating network between hospitals and public health services during pregnancy periods will be applicable in a community with different settings. With regard to the policy and clinical implications, establishment of a structure to support women with psychosocial problems from the start of pregnancy to after childbirth is beneficial.

## Conclusion

The current study proposed a multidisciplinary health service intervention program providing continuous support to women and their children from the start of pregnancy till after childbirth, and demonstrated its effectiveness in making women's mental health better in the postnatal period and help women and their children receive more services from public health nurses.

## Additional files

Additional file 1: Title and description of data: Characteristics of the multidisciplinary team of the Suzaka Program. (PDF 355 kb)

Additional file 2: Title and description of data: An example of case management provided by the multidisciplinary continuous support network. (PDF 372 kb)

Additional file 3: Title and description of data: Self-administered questionnaire for pregnant women when they turned in their pregnancy notification form in Suzaka City (English translation). (PDF 392 kb)

Additional file 4: Title and description of data: Self-administered questionnaire for women 3 and 4 months after delivery in Suzaka City (English translation). (PDF 345 kb)

Additional file 5: Title and description of data: The results of the psychosocial questionnaire of the intervention group’s participants at T1. (PDF 276 kb)

## Abbreviations

EPDS: Edinburgh Postnatal Depression Scale
PND: Pstnatal depression
SD: Standard deviation
