# Supplementary material for: Integrated mental health care in a multidisciplinary maternal and child health service in the community: the findings from the Suzaka trial
Source: BMC Pregnancy Childbirth. 2019 Feb 6;19:58. doi: 10.1186/s12884-019-2179-9 (PMC6364479; doi:10.1186/s12884-019-2179-9)
Supplement: Supplementary file 1 — Title and description of data: Characteristics of the multidisciplinary team of the Suzaka Program. (PDF 355 kb) [file 12884_2019_2179_MOESM1_ESM.pdf]

Additional file 1. Characteristics of the multidisciplinary team of the Suzaka Program

- Central coordination:

  The Suzaka public health office plays a central role in coordination.

- Clear mission statement and unambiguous rules of engagement:

  This system was based on the clear mission statement for perinatal care "Suzaka Perinatal Care Project" which started from 2014, and the mother and child care professionals in Suzaka City had been involved in the project.

- Inclusivity–ensuring all agencies and individuals gain ownership of the network.

  All professionals involved in the care of the mother and child in Suzaka City were included in the meetings and gained ownership of the network.

- Manageable size

  The number of mothers and children included in this program was manageable for public health nurses and related professionals providing cares (the population of Suzaka City was 51,549 in 2012; 51,234 in 2013; and 51,045 in 2014, while the number of the births were 369 in 2012, 350 in 2013, and 360 in 2014.) One public health nurse specifically selected for this program, 4 full-time public health nurses, and 10 part-time public health nurses supported the mothers and their children.

- Cohesion

  The meetings were developed with the aim of achieving network cohesion through joint finance arrangements, agreed care protocols and common targets (i.e., support for mother and child from the start of pregnancy).

- Ownership facilitated by formalized contracts and agreements

"Suzaka Perinatal Care Project" which is Suzaka City's multidisciplinary network for maternal and child health services facilitated by Suzaka City's public health centers was conducted after formal agreement by the related professionals.

- Leadership

  Public health nurses had active professional leadership in the meetings, and they could consult whether they should refer the mothers at risk of mental health problems.

- Avoidance of network domination by a professional elite or a particular organizational culture

  The meetings guaranteed equality between all participants, and they were invited to share their opinions freely.

- Response to the needs of network members

  The care plans for the mothers and their children were developed based on the discussions held at the meeting. The care plans were updated if necessary according to the discussion of the multidisciplinary meetings.

- Professionals in networks providing mandates to allow managers to manage and govern activities

  Professionals involved in the care of mother and child in Suzaka City provided mandates allowing public health nurses to manage the cases.
