# Supplementary material for: Integrated mental health care in a multidisciplinary maternal and child health service in the community: the findings from the Suzaka trial
Source: BMC Pregnancy Childbirth. 2019 Feb 6;19:58. doi: 10.1186/s12884-019-2179-9 (PMC6364479; doi:10.1186/s12884-019-2179-9)
Supplement: Supplementary file 2 — Title and description of data: An example of case management provided by the multidisciplinary continuous support network. (PDF 372 kb) [file 12884_2019_2179_MOESM2_ESM.pdf]

Additional file 2. An example of case management provided by the multidisciplinary continuous support network

(Individual information has been modified to protect the patient's privacy.)

History

Mrs. X was a 30-year-old woman in 20th week of gestation. She was first diagnosed with depression at age 25, and received medication treatment at a psychiatric clinic. However, treatment was stopped at the age of 27. She was a primipara.

Initial interview by the public health nurse

The initial interview conducted by the public health nurse at the time of submission of the pregnancy notification form revealed that the patient had been separated from the fetus' biological father 15 weeks into gestation. Informed consent was collected so as to allow development of a multidisciplinary care plan and provision of support.

The patient reported that she lived alone, felt quite lonely, and had no one to help her and her child after delivery. She had suffered from child abuse by her biological mother and had, therefore, not remained in contact with her. Her total Edinburgh Depression Scale score was 20, and she reported suffering from anxiety and depressive moods to the public health nurse. The public health nurse attentively listened to how the patient was feeling and what she had suffered from, particularly with regard to the points where the EDS's items were positive, and this attitude contributed to the development of a relationship with Mrs. X. The patient reported that she had decided to deliver her child, although she did not feel any attachment with her fetus.

The public health nurse diagnosed her with the following psychosocial problems:

- She was lacking emotional support.
- She had a psychiatric treatment history of depression, and had also been depressed during the pregnancy.
- She had lack of practical support after delivery.
- She had attachment problems with her fetus.

Care plan developed in the multidisciplinary meeting

The public health nurses presented this case at the multidisciplinary meeting, and the members developed a customized care plan.

The discussion on the care plan was as follows:

Comments from the psychiatrist

- She was at risk of depression and it was suggested that the public health nurses and other staff at the department of gynecology in Suzaka Hospital should recommend her to see her previous psychiatrist again.
- She seemed to have attachment problem with her fetus. Therefore, it may be beneficial for her that medical staff and public health nurses recognized her positive attitudes and praised them in order to enhance her competence of becoming a mother and her attachment with her fetus.
- She might demonstrate anxiety or report depressive mood. In such cases, it would be beneficial for her that the staff listens closely to what she has to say.
- She was at risk of postpartum depression and child maltreatment.

Information from the midwife and obstetrician

- She frequently asked for test results and complained about her health, and these could be caused by her anxiety.

Care plan during pregnancy

- The Staff would listen to her anxiety concerns thereby helping in improving her mental health.
- The gynecologist recommended that Mrs. X should see a psychiatrist again, and that her psychiatric treatment information should be shared via the public health nurses, provided the patient gave informed consent.
- The staff would encourage her to have a positive attitude toward child rearing so to enhance her competence as a mother and her attachment with her fetus.
- The midwives would discuss a birth plan with Mrs. X based on her psychosocial information.

The patient's case was followed-up at every multidisciplinary meeting. She was seen by her previous psychiatrist, and received cognitive behavior therapy at the outpatient clinic. The pediatrician and outpatient clinic nurse at the pediatric department in Suzaka prefectural hospital attended the multidisciplinary meeting to allow the patient to meet the staff that would see her and her child after delivery.

After delivery

She reported severe depressive mood post-delivery in the obstetric ward. As she had been thought to be at risk of postpartum depression, the obstetricians asked the psychiatrist to see her again. She was prescribed anti-depressant drugs, and she told the midwives about her level of attachment with her child. However, she also complained about experiencing anxiety regarding child care.

The multidisciplinary meeting held 1 week after her delivery

Comment from a psychiatrist

- As she was at risk of postnatal depression, her load of child care should be decreased through usage of postnatal day care. Additionally, her symptoms of depression and the child care status should be monitored.

Care plan of the midwives

- As monitoring of the patient's mental status and her care of the child was necessary, they advised her to make an appointment with the breast care outpatient clinic where she would be asked if she had any concerns regarding her condition or that of her child.

Care plan of the pediatrician

- He would check whether she has concerns regarding child care along with her child's medical examination to assess his/her health status at age 1 month. In addition, they would pay attention to her condition as well as the child's physical condition if her child is presented to the pediatric outpatient clinic with some diseases.

Care plan of the public health nurse

- They would follow-up when the infant is 3–4 months, 7–8 months, 1 and a half year, and 3 year old, followed by toddler medical examinations. They would suggest her to use the postnatal day care.
- They would carry out home visits or telephone frequently to support her and her child if necessary.

As a result of these multidisciplinary meetings, the mother and her child, who were at risk of psychosocial problems, received continuous support from the related professionals, i.e.,

obstetricians, midwives, nurses, pediatricians, psychiatrists, and public health nurses.
