# Supplementary material for: Integrated mental health care in a multidisciplinary maternal and child health service in the community: the findings from the Suzaka trial
Source: BMC Pregnancy Childbirth. 2019 Feb 6;19:58. doi: 10.1186/s12884-019-2179-9 (PMC6364479; doi:10.1186/s12884-019-2179-9)
Supplement: Supplementary file 3 — Title and description of data: Self-administered questionnaire for pregnant women when they turned in their pregnancy notification form in Suzaka City (English translation). (PDF 392 kb) [file 12884_2019_2179_MOESM3_ESM.pdf]

Additional file 3. Self-administered questionnaire for pregnant women when they turned in their pregnancy notification form in Suzaka City (English translation)

To pregnant mother

Congratulation on your pregnancy! Suzaka city supports mothers during their perinatal periods. Please fill out the following questions only if do not cause you any difficulties.

I. The first few questions are about your life.

Please tick the relevant box inquiring about who you have been living with?

□Husband □partner □child(ren) [number of your children if any____________], □biological father □biological mother □mother-in-law □father-in-law

□your brother or sister

□others_____________

How many people in your family? _____________________

1. Do you smoke?

Not at all; thus far; ceased before this pregnancy; ceased after this pregnancy; smoking now

How many cigarettes do you smoke in a day? _____________ cigarettes

2. Are there any members in your family that smoke at your home?

□Yes (______________________)

□No

How many cigarettes does he/she/ they smoke, if any? _____________ cigarettes

3. Do you drink?

Not at all; thus far; ceased before this pregnancy; ceased after this pregnancy; drinking now

4. Have you been taking any medications?

□Yes (______________________)

□No

5. Please enter your height and weight.

（　　　　　）cm、（　　　　　）kg

II. This section focuses on your pregnancy, delivery, and child care.

1. How did you feel when you found out that you were pregnant?

□I was happy

□It was not expected, but I was happy.

□It was not expected, so I did not know what to do.

□I was at a loss.

□I did not feel anything special

2. Do you have someone to help you through your antenatal and postnatal periods?

□Yes □No

3. Do you feel free to talk with your partner about what you have gone through?

□Yes □No

4. Do you feel free to talk with your biological mother about what you have gone through?

□Yes □No

5. Do you have someone besides your partner and biological mother to talk with about

what you have gone through?

Who is he or she? (______________________)

6. Do you have a difficult time in life or are you financially anxious?

□Yes □No

7. Have you experienced a stillbirth, misbirth, or your child’s death within a year?

□Yes □No

8. Have you ever seen a psychologist or psychiatrist for any psychological problems?

□Yes □No

9. Have you ever received treatment for any physical disease?

□Yes □No

10. Have you ever experienced insomnia, irritability, tearfulness, apathy lasting for at least two weeks within a year?

□Yes □No

11. Do you have any concerns about your pregnancy or delivery?
