# Supplementary material for: Integrated mental health care in a multidisciplinary maternal and child health service in the community: the findings from the Suzaka trial
Source: BMC Pregnancy Childbirth. 2019 Feb 6;19:58. doi: 10.1186/s12884-019-2179-9 (PMC6364479; doi:10.1186/s12884-019-2179-9)
Supplement: Supplementary file 4 — Title and description of data: Self-administered questionnaire for women 3 and 4 months after delivery in Suzaka City (English translation). (PDF 345 kb) [file 12884_2019_2179_MOESM4_ESM.pdf]

Additional file 4. Self-administered questionnaire for women 3 and 4 months after delivery in Suzaka City (English translation)

How have you been in these days 3 to 4 months post-delivery?

Suzaka City administered a questionnaire to women in their postpartum period. We would like to use your answers for our services as a reference. Please answer the following questions.

1. What kind of services have you received?

   Please check the box.

□Interview by public health nurse at the time of submitting the pregnancy notification form

□Counseling by public health nurse during pregnancy

□Maternity seminar

□Postnatal care service (provided by Suzaka prefectural hospital or a maternity center)

□Neonatal home visit by a public health nurse or a midwife

□Telephone counseling by the public health center

□Counseling regarding child care or measuring the baby's weight at the public health center

□Home help service provided by a private company

□Others

In addition to the questions above, the EPDS was implemented.
