# Supplementary material for: Integrated mental health care in a multidisciplinary maternal and child health service in the community: the findings from the Suzaka trial
Source: BMC Pregnancy Childbirth. 2019 Feb 6;19:58. doi: 10.1186/s12884-019-2179-9 (PMC6364479; doi:10.1186/s12884-019-2179-9)
Supplement: Supplementary file 5 — Title and description of data: The results of the psychosocial questionnaire of the intervention group’s participants at T1. (PDF 276 kb) [file 12884_2019_2179_MOESM5_ESM.pdf]

**Additional file 5. The results of the psychosocial questionnaire of the intervention group's participants at T1**

| | Missing | Number |
|---|---|---|
| Number of the family living with | | |
| 1 | | 61 |
| 2 | | 67 |
| 3 | | 40 |
| 4 | | 21 |
| 5 | | 12 |
| 6 | | 5 |
| 7 | | 4 |
| Persons who live with | | |
| partner | 1 | 205 |
| child | | |
| 0 | | 91 |
| 1 | | 84 |
| 2 | | 25 |
| 3 or more | | 10 |
| partner's father | | 26 |
| partner's mother | | 33 |
| mother | | 25 |
| father | 1 | 19 |
| siblings | | |
| 0 | | 196 |
| 1 | | 10 |
| 2 or more | | 4 |
| others | | 15 |
| Smoking | | |
| Non-smoker also before pregnancy | | 169 |
| Stopped after pregnancy | | 37 |
| Yes | | 4 |
| Drinking | 1 | |
| Non-drinker also before pregnancy | | 104 |

| | Missing | Number |
|---|---|---|
| Practical supports during pregnancy and after delivery | 2 | |
| Yes | | 208 |
| No | | 2 |
| Partner's emotional supports | 2 | |
| Yes | | 202 |
| No | | 2 |
| Mother's emotional supports | 4 | |
| Yes | | 197 |
| No | | 3 |
| Neither | | 6 |
| Others' emotional supports | 4 | |
| Yes | | 194 |
| No | | 12 |
| Experience of misbirth, stillbirth, or baby death within a year after delivery | 2 | |
| Yes | | 48 |
| No | | 160 |
| For those who have an experience of delivery | | |
| How had your condition been during antenatal and postnatal periods? | 85 | |
| well | | 86 |
| irritated | | 16 |
| felt anxiety | | 12 |
| depressed | | 3 |
| felt sick | | 1 |
| others | | 7 |
| Have you ever had some physical or fertile problems during pregnancy? | 88 | |
| Yes | | 21 |
| No | | 101 |
| How do you feel when you knew you had become pregnant? | 4 | |
| very happy | | 142 |
| unexpected but happy | | 56 |

| | | |
|---|---|---|
| Stopped after pregnancy | | 105 |
| Medication | 4 | |
| Yes | | 24 |
| No | | 182 |
| Height | | 157.83 |
| Body weight | | 51.46 |
| BMI | | 20.5 |
| Economic funk | 4 | |
| Yes | | 68 |
| No | | 138 |

| | | |
|---|---|---|
| unexpected and confused | | 5 |
| baffled | | 2 |
| others | | 1 |
| Past psychiatric treatment history | 37 | |
| Yes | | 13 |
| No | | 160 |
| Past physical illness treatment history except for pregnancy | 37 | |
| Yes | | 17 |
| No | | 156 |
| | Mean | SD |
| EPDS total score | 3.7 | 2.83 |

EPDS indicates Edinburgh Postnatal Depression Scale. SD indicates standard deviation.
